# Jahn-Teller effect and features of divalent copper ion behavior in multicomponent borate crystals

**DOI:** 10.1038/s41598-024-66557-1

**Published:** 2024-07-09

**Authors:** Roman Minikayev, Aleksandr Prokhorov, Jan Lančok, Andrey Prokhorov

**Affiliations:** 1 Institute of Physics PAS, al. Lotnikow 32/46, 02-668 Warsaw, Poland; 2 A. A. Galkin Donetsk Physico-Technical Institute, R. Luxemburg 72, Donetsk, 83114 Ukraine; 3 Institute of Physics AS CR, Na Slovance 2, 18221 Prague, Czech Republic; 4 Institute of Plasma Physics of the Czech Academy of Sciences, U Slovanky 2525/1a, 182 00 Prague, Czech Republic

**Keywords:** Rare-earth borates, EPR spectroscopy, Ligand hyperfine structure, Jahn-Teller effect, Chemistry, Materials science, Physics

## Abstract

Crystals of YGa_3_(BO_3_)_4_, YAl_3_(BO_3_)_4_, EuGa_3_(BO_3_)_4_ and EuAl_3_(BO_3_)_4_ with copper alloy were studied by electron paramagnetic resonance and X-ray diffraction analysis. The lattice parameters and coordinates of copper-doped boron atoms were determined. The study of EPR spectra showed that copper is in the divalent state and replaces aluminum ions with C_2_ node symmetry. In YAl_3_(BO_3_)_4_:Cu crystals, a ligand structure exists due to the interaction of copper electrons with yttrium nuclei. The parameters of the spin Hamiltonian describing the behavior of the Cu^2+^ spectrum have been determined. The deviation of the *Z*-axis spectra from the C_3_ axis by 54(1)° is due to Jahn-Teller vibronic interaction and monoclinic distortion. In the EuGa_3_(BO3_)4_ crystal, a new spectrum 2 was found, which also belongs to divalent copper but is observed at an excited state 31 cm^−1^ away from the ground state. Above 70 K, an isotropic EPR line with a width of 450 Gs, *g* = 2.1, appears and exists up to room temperature.

## Introduction

According to the Jahn-Teller theorem^1^, in a state with electronic degeneracy, the nuclear configuration of a nonlinear multi-atom system is unstable due to nuclear shifts that remove this degeneracy. This effect is observed in EPR spectroscopy of several ions that have orbital ground state degeneracy. The most studied state is that of the divalent copper ion in octahedral environments^2,3^. In a regular octahedron, deformation along the fourth-order axis occurs to remove the orbital degeneracy. Since there are three such axes, distortions in three directions can occur with equal probability, and thus three magnetically nonequivalent spectra with the same parameters are observed. These three states are separated by a barrier, which is overcome by tunneling or thermal excitation. However, manifestations of the Jahn–Teller effect can also be observed in an octahedron initially deformed by slight additions of a weakly symmetric crystal field, as described in^4^. The properties of such systems are poorly known. Suitable borates are those with the general formula *RM*_3_(BO_3_)_4_, where *R* is a rare earth ion or yttrium, *M*-Al^3+^, Ga^3+^ or trivalent iron group ions. These crystals have two nodes which an admixture ion can occupy. The node occupied by the rare earth ion has trigonal symmetry (D_3_) and the node containing Al^3+^, and Ga^3+^ has C_2_ symmetry. As the studies of the EPR spectra of substitutional ions Al^3+^, and Ga^3+^^5–17^ have shown, their parameters are close to trigonal. Therefore it is possible to observe the Jahn-Teller effect in borate crystals.

This paper presents the results of a study on divalent copper ions introduced into EuAl(Ga)_3_(BO_3_)_4_ and YAl(Ga)_3_(BO_3_)_4_ crystals. This work aims to detect and investigate the spectra of divalent copper that replaces Al(Ga) ions. These ions are located in a slightly deformed octahedron of oxygen ions with nodal symmetry C2. Despite the low symmetry, the influence of the Jahn-Teller effect is evident, leading to new unusual properties. The study was performed by EPR over a wide temperature range, from helium to room temperature.

## Results

### Crystal structure features

Crystals of the borate family *RM*_3_(BO_3_)_4_, where *R* is a rare earth metal or yttrium and *M* are the trivalent ions Al, Ga, Fe, Sc, Cr, crystallize in the huntite structure CaMg_3_(CO_3_)_4_ with space group *R*32. The unit cell parameters and atomic positions of some of these crystals are given in Table 1.

**Table 1:** The crystallographic parameters of Cu ions doped YGa_3_(BO_3_)_4_, EuGa_3_(BO_3_)_4_, YAl_3_(BO_3_)_4_ and EuAl_3_(BO_3_)_4_ crystals.

Sample name							
YGa_3_(BO_3_)_4_: 0.2% Cu				EuGa_3_(BO_3_)_4_: 0.2% Cu			
Lattice parameters							
*a* = 9.4533(1) Å *c* = 7.4606(1) Å				*a* = 9.46860(7) Å *c* = 7.47222(7) Å			
Atomic positions							
Name	*x*	*y*	*z*	Name	*x*	*y*	*z*
Y	0	0	0	Eu	0	0	0
Al/Cu	0.55064(8)	0	0	Al/Cu	0.5520(1)	0	0
B(1)	0	0	0.5	B(1)	0	0	0.5
B(2)	0.4535(9)	0	0.5	B(2)	0.4547(9)	0	0.5
O(1)	0.8577(5)	0	0.5	O(1)	0.8568(5)	0	0.5
O(2)	0.5919(5)	0	0.5	O(2)	0.5891(4)	0	0.5
O(3)	0.4521(5)	0.1470(4)	0.5153(6)	O(3)	0.4591(5)	0.1518(4)	0.5119(6)

Sample name							
YAl_3_(BO_3_)_4_: 0.1% Cu^4^				EuAl_3_(BO_3_)_4_: 0.1% Cu^4^			
Lattice parameters							
*a* = 9.28022(4) Å *c* = 7.22747(4) Å				*a* = 9.30832(8) Å *c* = 7.26947(8) Å			
Atomic positions							
Atom	*x*	*y*	*z*	Atom	*x*	*y*	*z*
Y	0	0	0	Eu	0	0	0
Al/Cu	0.5545(2)	0	0	Al/Cu	0.5520(4)	0	0
B(1)	0	0	0.5	B(1)	0	0	0.5
B(2)	0.4478(8)	0	0.5	B(2)	0.4188(32)	0	0.5
O(1)	0.8595(4)	0	0.5	O(1)	0.8747(10)	0	0.5
O(2)	0.5823(5)	0	0.5	O(2)	0.5604(13)	0	0.5
O(3)	0.4476(4)	0.1501(4)	0.5206(4)	O(3)	0.4442(9)	0.1509(9)	0.5224(9)

**Figure 1:**
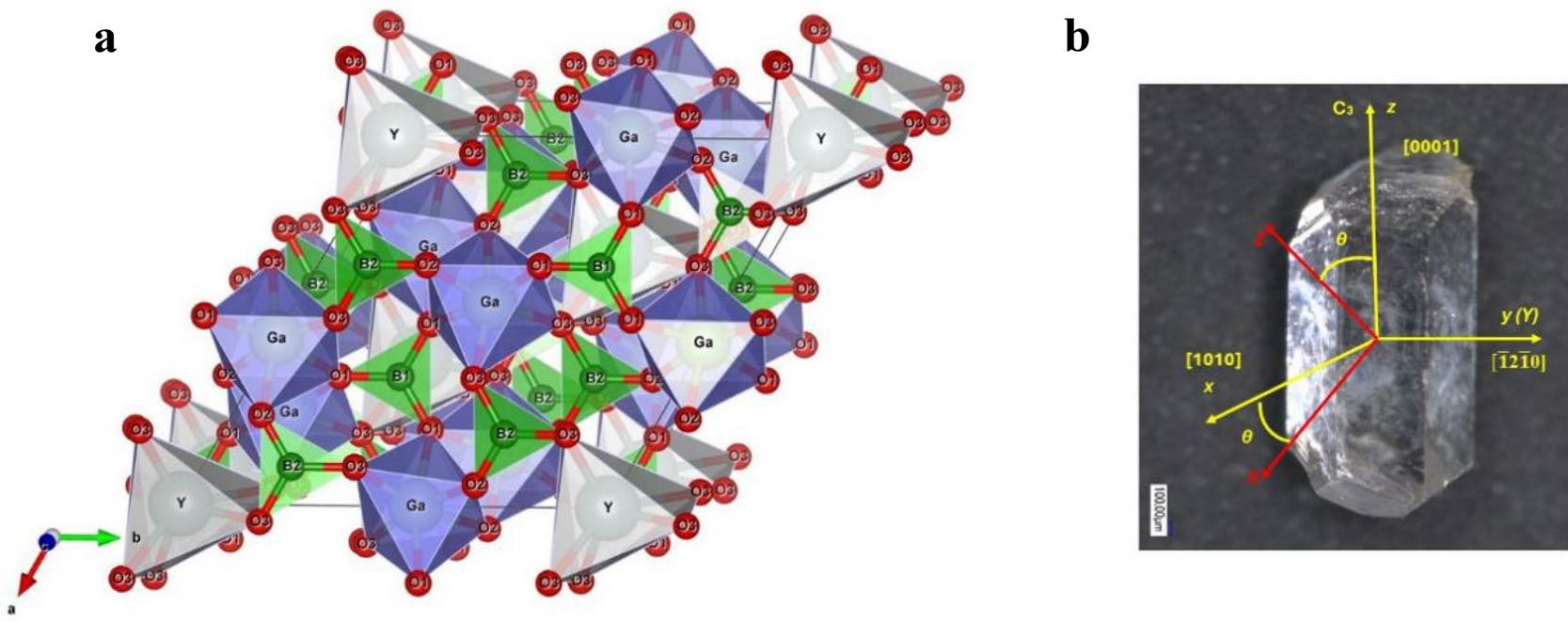
(**a**) A fragment of the crystal structure of YGa_3_(BO_3_)_4_. The Y^3+^ ion is in a prism with oxygen O_3_ ions in its peaks. Gallium or aluminum ions are in the deformed octahedra of oxygen ions O_1_, O_2_, O_3_. (**b**) Habitus of YGa_3_(BO_3_)_4_ crystal in the shape of an elongated hexagonal prism. The figure shows the crystal axes and the directions of the *X*, *Y* and *Z* axes of the EPR spectrum of the Cu^2+^ ion. The *Z* and *X* axes lie in the ($\overline{1 }$**2**$\overline{1 }$**0)** plane and the *Y* axis is perpendicular to this plane and coincides with the second-order crystal axis [$\overline{1 }$**2**$\overline{1 }$**0].**

The unit cell of *R*(Al/Ga)_3_(BO_3_)_4_ contains *z* = 3 formula units. The coordinating polyhedra *R*^3+^, Al^3+^ and B^3+^ are trigonal prisms, octahedra and triangles, respectively, formed by oxygen ions. The crystal structure of *R*(Al/Ga)_3_(BO_3_)_4_ borate is shown in Fig. 1a, b shows the habitus of YGa_3_(BO_3_)_4_ crystal which is typical for *R*Al_3_(BO_3_)_4_ and *R*Ga_3_(BO_3_)_4_ crystals in the shape of an elongated hexagonal prism.

### XRD investigations

The structure of YGa_3_(BO_3_)_4_: 0.2% Cu, EuGa_3_(BO_3_)_4_: 0.2% Cu, YAl_3_(BO_3_)_4_: 0.1% Cu, EuAl_3_(BO_3_)_4_: 0.1% Cu crystals were investigated by the XRD method. All samples are crystallized in the huntite type structure. The crystals itself do not contain any impurity phases but among of the YGa_3_(BO_3_)_4_: 0.2% Cu crystallites the β-Ga_2_O_3_ crystals was detected. The gallium oxide was separated of borate crystallites but a small amount ~ 2.5 at % are left. The presence of additional phases did not influence significantly the crystallographic analysis results.

**Figure 2:**
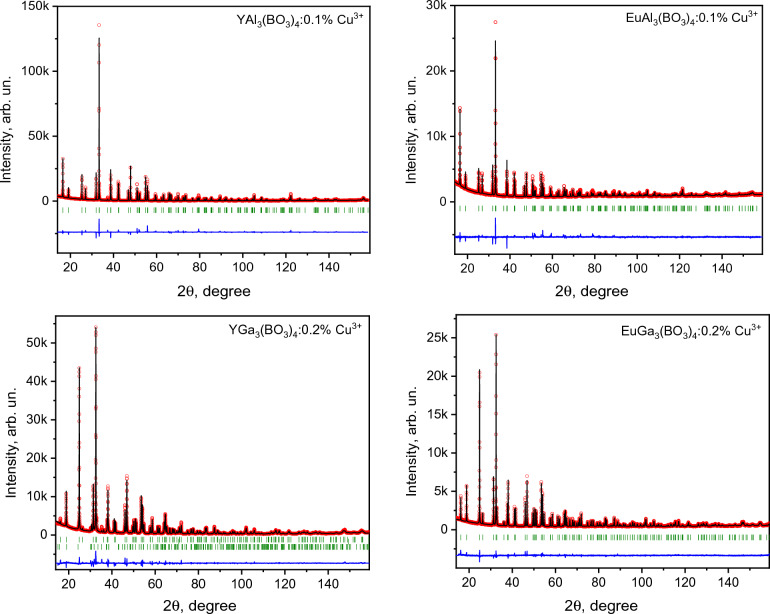
Rietveld refinement results for YGa_3_(BO_3_)_4_: 0.2% Cu, EuGa_3_(BO_3_)_4_: 0.2% Cu, YAl_3_(BO_3_)_4_: 0.1% Cu^4^ and EuAl_3_(BO_3_)_4_: 0.1% Cu^4^. Open red circles are experimental points, black solid lines are refined pattern profile, vertical green bars are Bragg reflection positions, lowest bars on YGa_3_(BO_3_)_4_: 0.2% Cu pattern are belonged to minor beta gallium oxide phase.

The crystal structure of investigated in this paper aluminum-based borates was published earlier in ref.^4^. The initial crystallographic model of the gallium-based borates used in the Rietveld analysis was based on models obtained in ref ^15^. and^18^. The refined unit cells parameters and atomic positions of gallium and aluminum borates are placed in Table 1, and graphical results of the fitting of YGa_3_(BO_3_)_4_: 0.2% Cu, EuGa_3_(BO_3_)_4_: 0.2% Cu as well as earlier published YAl_3_(BO_3_)_4_: 0.1% Cu and EuAl_3_(BO_3_)_4_: 0.1% Cu^4^ are shown in Fig. 2. The obtained lattice parameters of YGa_3_(BO_3_)_4_: 0.2% Cu and their ratio (0.78921(3)) are slightly bigger than those earlier reported in ref ^15^. (0.78822(3)) and^10^ (0.78893(3)). This effect probably can be caused by defects in the crystal. In other hand the unit cell parameters obtained for EuGa_3_(BO_3_)_4_: 0.2% Cu are in good agreement with data published in ref.^19^. In particular the *c*/*a* value 0.78916(2) is close to value 0.78944(3) reported for Tb^3+^ doped EuGa_3_(BO_3_)_4_^18^. The refined atomic position values have no significant differences with initial crystallographic models. In comparison with the parameters of unit cells obtained for aluminum borates^4^, the replacement of aluminum ions with gallium ion leads both to an increase in lattice parameters and to an increase in their ratio, which is a direct consequence of the difference in ion sizes. Meanwhile, the XRD results obtained in both studies confirm the declared crystallographic characteristics of the investigated crystals.

### EPR spectrum of Cu^2+^ in EuAl_3_(BO_3_)_4_:0.1% Cu and YAl_3_(BO_3_)_4_: 0.1% Cu crystals

**Figure 3:**
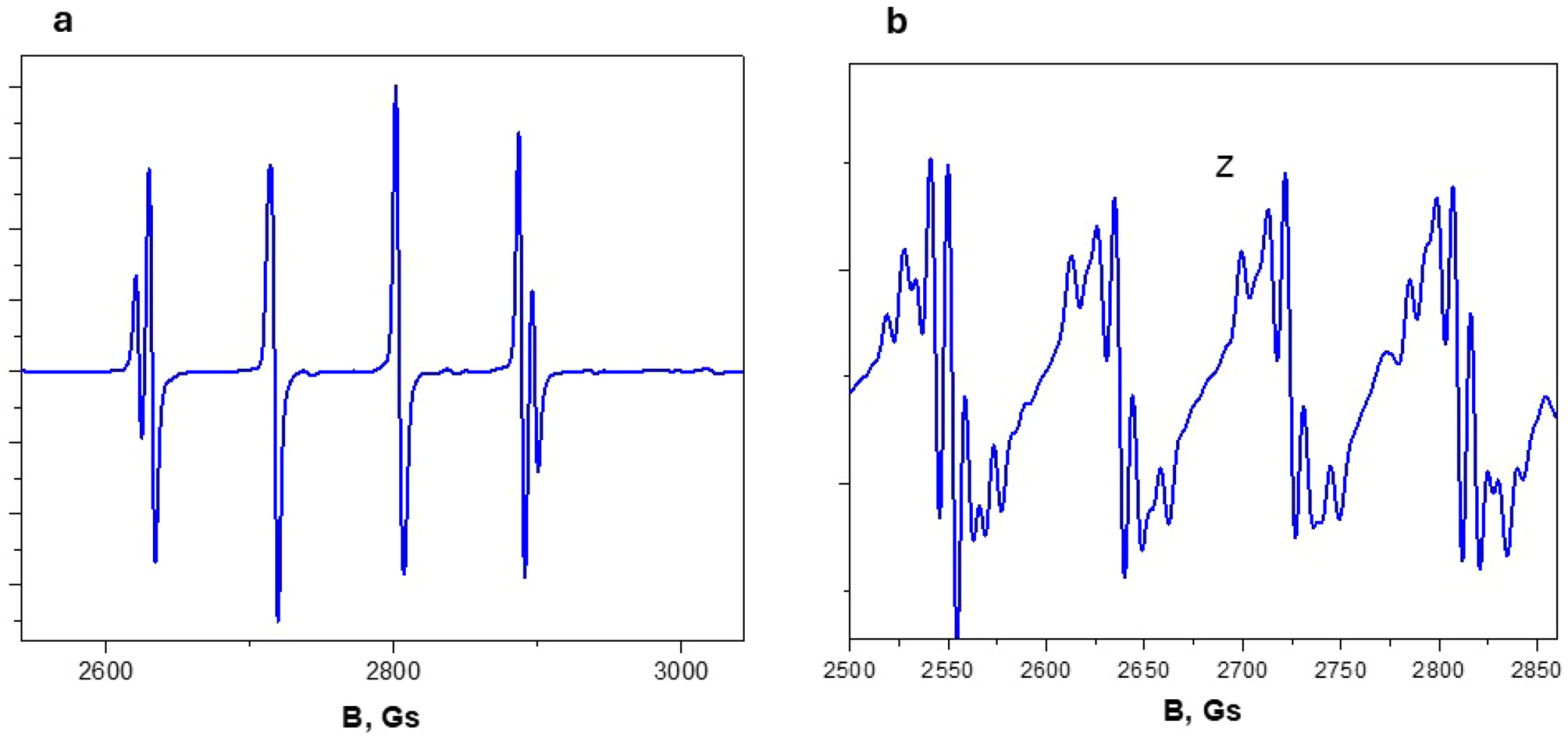
(**a**) EPR spectrum of Cu^2+^ ion in EuAl_3_(BO_3_)_4_:Cu crystal. The isotopic structure caused by the isotopes ^63^Cu and ^65^Cu is visible.(b) EPR spectrum of Cu^2+^ ion in YAl_3_(BO_3_)_4_:Cu crystal. Besides the isotopic structure caused by ^63^Cu and ^65^Cu isotopes, other lines of ligand structure caused by interaction with yttrium ion nuclei are observed.

In copper oxide (CuO) doped crystals, the EPR spectrum of divalent copper was found to have characteristic features inherent to this particular ion. Figure 3a, b shows the spectra at 15 K in the crystal—EuAl_3_(BO_3_)_4_:Cu and a similar record in the crystal YAl_3_(BO_3_)_4_:Cu when the magnetic field is directed along the *Z* axis of the spectrum. We observe a hyperfine structure caused by two copper isotopes with the same core spin (*I* = 3/2), different core magnetic moments of 65Cu(− 0.220), 63Cu(− 0.204) and different contents of 65Cu(31%) and 63Cu(69%). In the EuAl_3_(BO_3_)_4_:Cu crystal, the isotopic structure is visible. In the YAl_3_(BO_3_)_4_:Cu crystal, each line has an additional cleavage. From the angular dependence, the directions of the spectral axes concerning the crystal facet were determined. The *Z* axis of the spectrum is shifted away from the C_3_ axis. Three magnetically non-equivalent spectra are observed in the crystals, which have the same parameters and repeat at 1200 rotation about the C_3_ axis. The *Z* and *X* axes lie in the ($\overline{1 }$**2**$\overline{1 }$**0)** plane and the *Y* axis, which is the second-order axis, is perpendicular to this plane.

Qualitatively, the EPR spectra in both crystals are identical, but the numerical characteristics of the spectra differ. The observed EPR spectrum of the Cu^2+^ ion in the whole temperature region investigated is described with sufficient accuracy by the rhombic spin Hamiltonian^2^.

1 $$ \hat{H} = {\upbeta }{\mathbf{B}}g\hat{S} + \hat{S}A\hat{I} $$

where *g*-the spectroscopic splitting tensor; *A*-the hyperfine interaction tensor; *β*- Bohr magneton; *B*-the magnetic field induction vector; *Ŝ*-the electron spin operator; *I*-the nuclear spin operator. The parameters of the spectra of both crystals are given in Table 2.

**Table 2:** Parameters of the spin hamiltonian describing the EPR spectrum in Cu doped YAl_3_(BO3)_4_, EuAl_3_(BO_3_)_4_, YGa_3_(BO_3_)_4_, EuGa_3_(BO_3_)_4_ crystals.

Crystal	*g*_*x*_	*g*_*y*_	*g*_*z*_	*Ax*, MHz	*A*_*y*_, MHz	*A*_*z*_, MHz	θ, °
YAl_3_(BO_3_)_4_: 0.1% Cu-15 K	2.122(2)	2.132(2)	2.500(2)	66.3(3)	55.8(3)	320.04(2) -Cu65 300.23(2) -Cu63	53.1
EuAl_3_(BO_3_)_4_: 0.1% Cu-15 K	2.111(2)	2.106(2)	2.428(2)	68.3(3)	57.1(3)	304.27(2) -Cu65 291.36(2) -Cu63	54.2
YGa_3_(BO_3_)_4_: 0.2% Cu-20 K	2.104(2)	2.099(2)	2.454(2)	100.1(3)	85.1(3)	271.3(2)	54(1)
EuGa_3_(BO_3_)_4_: 0.2% Cu-20 K Spectr 1	2.162(2)	2.225(2)	2.446(2)	224.5(3)	135.8(3)	257.8(2)	54(1)
EuGa_3_(BO_3_)_4_: 0.2% Cu-20 K Spectr 2	2.138(2)	2.121(2)	2.399(2)	259.1(3)	103.8(3)	379.5(2)	54(1)

For clarity, the faceting of the aluminum borate crystal and the direction of the spectra axes are shown in Fig. 1. The Y axis of the Cu^2+^ ion spectrum coincides with the second order axis [$\overline{1 }$ **2** $\overline{1 }$ **0]** and is perpendicular to the crystal surface. The *X* and *Z* axes lie in the plane **(**$\overline{1 }$** 2** $\overline{1 }$ **0)** and do not coincide with the crystallographic axes. The values of the angles of deviation of the *Z* axis from the C_3_ axis are given in Table 2.

**Figure 4:**
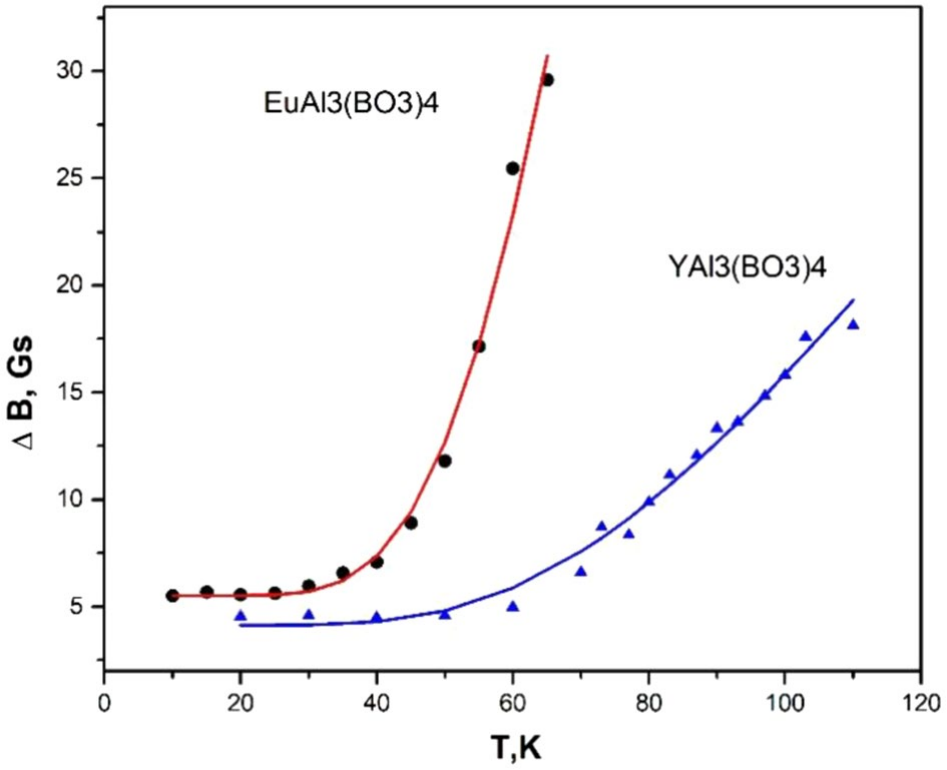
The temperature dependence of the EPR linewidths in YAl_3_(BO_3_)_4_:Cu and EuAl_3_(BO_3_)_4_:Cu crystals.

In the X region, the EPR linewidth at 15 K in the EuAl_3_(BO_3_)_4_:Cu crystal is 4 Gs in the *Z* orientation and 5.5 Gs in the *X* orientation. Under the same conditions, in the YAl_3_(BO_3_)_4_ crystals, the linewidth is 4.7 Gs in the *Z* orientation and 7 Gs in the *X* orientation. In the *Q*-band, the linewidths are on the order of 30 Gs, and fine features of the spectra are not observed. With increasing temperature above 20 K, the spectral lines broaden due to an increase in the spin–lattice relaxation rate. Figure 4 shows the temperature dependence of the EPR linewidths in both crystals, which can be interpreted using the exponential dependence $\Delta B = c_{0} + c_{1} *exp\left( { - c_{2} /T} \right).$

**Figure 5:**
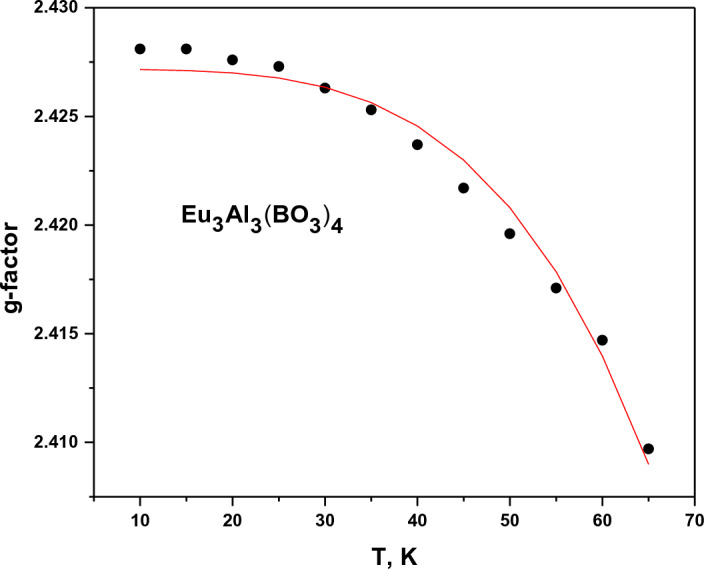
Temperature dependence of *g*-factor EPR spectrum of Cu^2+^ ion in EuAl_3_(BO_3_)_4_:Cu crystal.

In the EuAl_3_(BO_3_)_4_:Cu crystal: *c*_0_ = 5.4(4); *c*_1_ = 1.7(5)∙10^3^; *c*_2_ = 271(21) K = 187(14) cm^−1^. In the YAl_3_(BO_3_)_4_:Cu crystal: *c*_0_ = 4.1(4); *c*_1_ = 2.0(5)∙10^2^; *c*_2_ = 284(28) K = 196(19) cm^−1^. The spin–lattice relaxation rate is more effective in the EuAl_3_(BO_3_)_4_:Cu crystal, but the exponential dependence indices are almost the same in both crystals. Simultaneously with the broadening of the lines with increasing temperature, a decrease in *g*_*z*_ is observed, as shown in Fig. 5.

### EPR spectrum of Cu^2+^ in YGa_3_(BO_3_)_4_:0.2% Cu and EuGa_3_(BO_3_)_4_:0.2% Cu crystals

**Figure 6:**
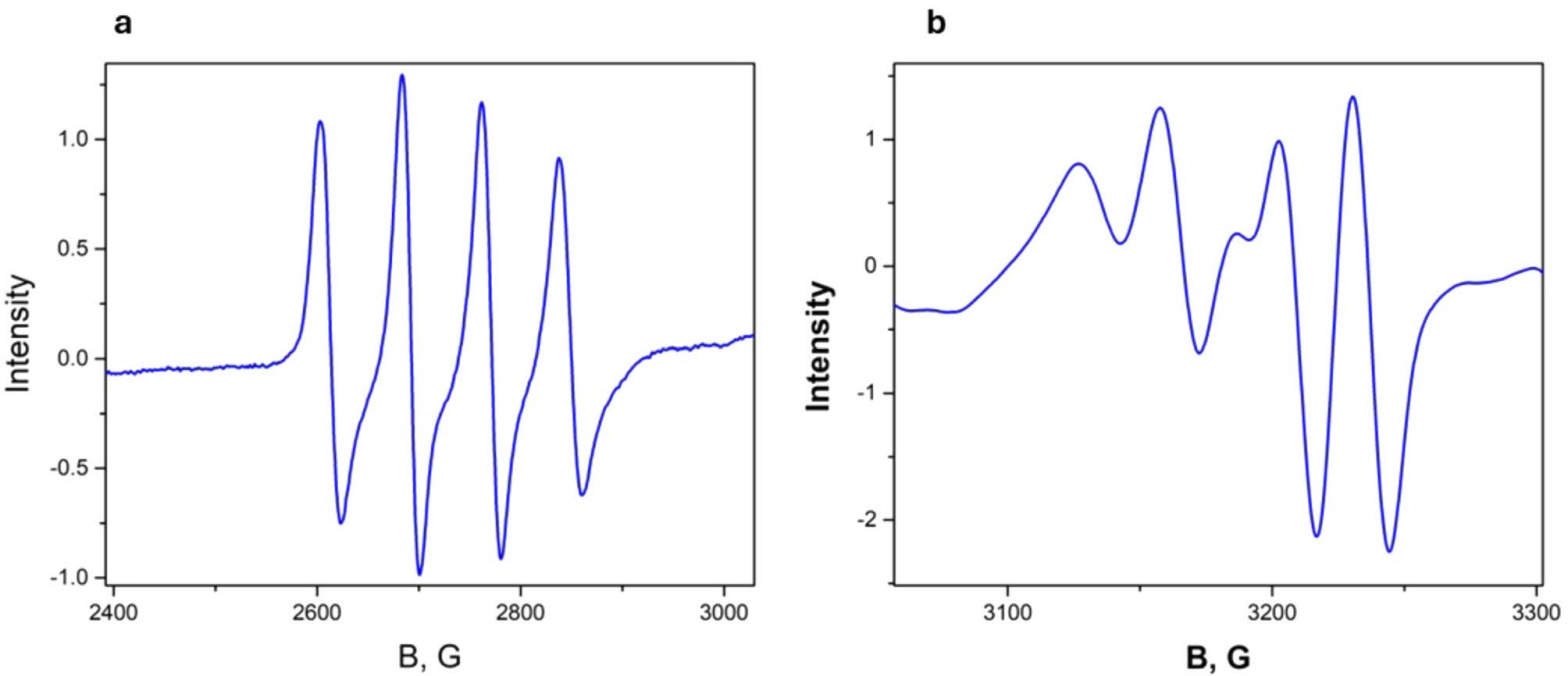
(**a**) EPR spectrum of Cu^2+^ ion in YGa_3_(BO_3_)_4_:Cu crystal at 20 K in *Z* orientation. (**b**) EPR spectrum of Cu^2+^ ion in YGa_3_(BO_3_)_4_:Cu crystal at 20 K in *X* orientation.

In YGa_3_(BO_3_)_4_:Cu crystals, it was observed that the EPR spectrum of divalent Cu consists of four lines of hyperfine structure, which is due to the nuclear momentum of Cu with spin *I* = 3/2. Unlike the EPR spectra of divalent Cu in aluminoborate crystals^4^, the isotopic structure due to two Cu isotopes is not observed due to the considerable line width. In the YGa_3_(BO_3_)_4_:Cu crystal, the line width is 18 Gs. Figure 6a shows the spectrum recording in *Z* orientation and Fig. 6b in *X* orientation at *f* = 9385 MHz at 20 K.

Three magnetically non-equivalent spectra are observed in the crystals, which have the same parameters and are repeated at 1200 rotations about the C_3_ axis. The *Z* and *X* axes lie in the **(**$\overline{1 }$** 2** $\overline{1 }$ **0)** plane and the *Y* axis, which is the second-order axis, is perpendicular to this plane. The *Z* axis is offset from the C_3_ axis by 54(1) degrees. The crystal habitus with the directions of the crystal axes and the EPR spectrum axes are shown in Fig. 1b.

The EPR spectrum of Cu^2+^ ion can be described by the above rhombic spin Hamiltonian^2^.

**Figure 7:**
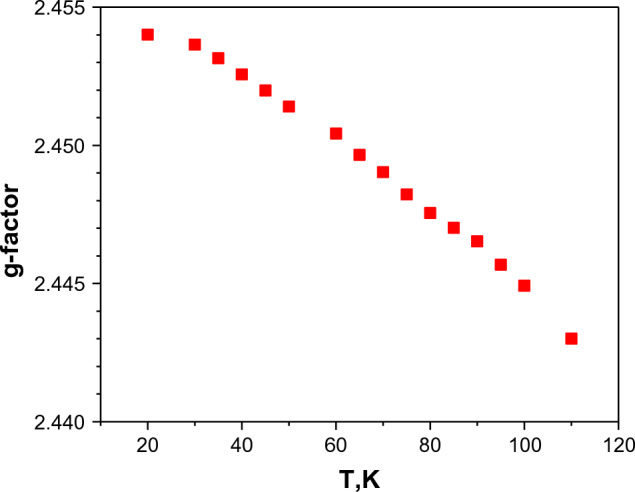
Change of g-factor of Cu^2+^ ion in YGa_3_(BO_3_)_4_:Cu crystal as a function of temperature.

The line width does not change with increasing temperature, but the g-factor decreases slightly Fig. 7 and the hyperfine splitting constant increases.

**Figure 8:**
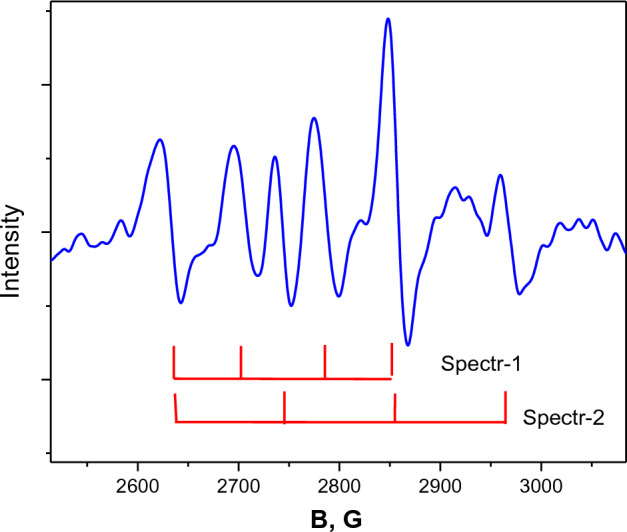
EPR spectrum of Cu^2+^ ion in EuGa_3_(BO_3_)_4_:Cu crystal at 40 K in *Z* orientation. Two spectra of approximately the same intensity are observed.

**Figure 9:**
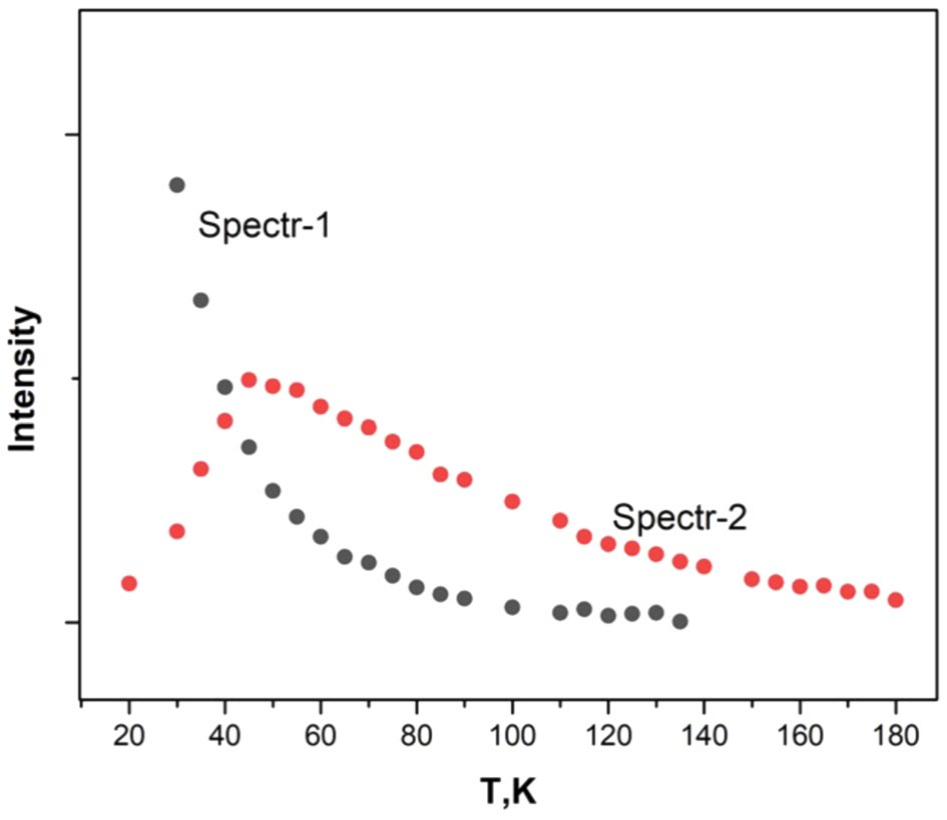
Temperature dependence of Spectr-1 and Spectr-2 peak intensities in EuGa_3_(BO_3_)_4_:Cu crystal.

**Figure 10:**
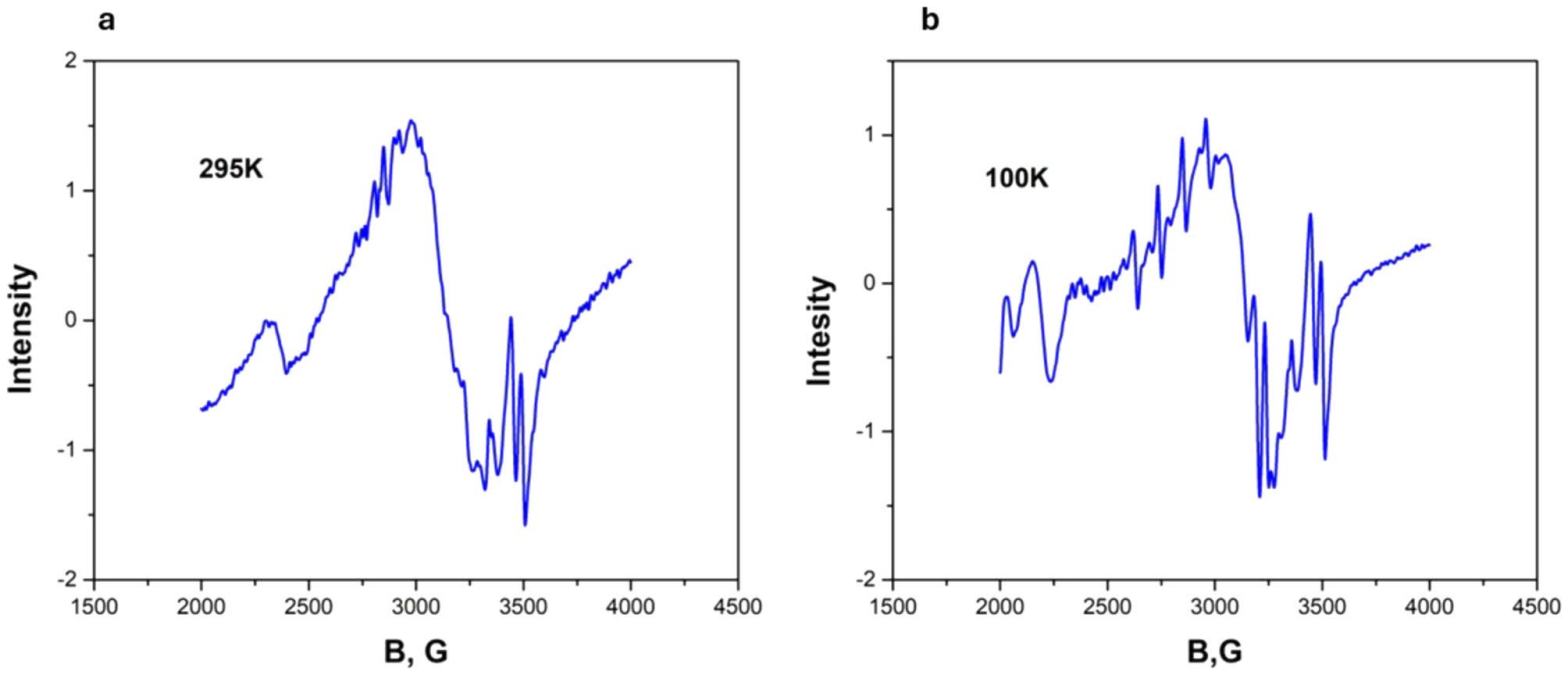
(**a**) EPR line with a width of 450 G and a *g*-factor of 2.1 at 295 K in a EuGa_3_(BO_3_)_4_:Cu crystal. (**b**) EPR line with a width of 450 G and a *g*-factor of 2.1 at 100 K in the EuGa_3_(BO_3_)_4_:Cu crystal the ultra-thin structure of Spectr-2 is visible.

Two spectra belonging to divalent copper ion are observed in EuGa_3_(BO_3_)_4_:Cu crystal, the intensity of the first one (*g*_*z*_ = 2.445, *g*_*x*_ = 2.158, *A*_z_ = 257.8 MHz *A*_x_ = 225 MHz) decreases with heating from 4 to 140 K, the intensity of the second (*g*_*z*_ = 2.399, *g*_*x*_ = 2.138, *A*_*z*_ = 379.5 MHz, *A*_*x*_ = 274.1 MHz) increases up to 45 K, decreases with further heating and is observed up to 180 K. Figure 8 shows a recording of the EPR spectrum at *T* = 40 K, which shows both spectra of approximately the same intensity. Figure 9 shows the temperature dependence of the peak intensity of the first and second spectra. The maximum intensity of the second spectrum is observed around 45 K. The *Z* axes of both spectra are tilted away from the C_3_ axis by 54(1) degrees. Above 70 K, an isotropic EPR line with a width of 450 Gs (*g* = 2.1) appears and exists up to room temperature. In addition to the broad line, a hyperfine structure of the second spectrum is observed. On further heating, the intensity of the hyperfine structure decreases and is no longer visible at 290 K Fig. 10a,b. Lines from uncontrolled impurities entering the crystal from the oxides used in the filling composition are also observed in the record.

## Discussion

### YAl_3_(BO_3_)_4_:0.1% Cu and EuAl_3_(BO_3_)_4_:0.1% Cu

As already mentioned, the aluminoborate lattice has two nodes into which impurity ions can enter—trivalent yttrium and trivalent aluminium. The ions Cr^3+^, Ti^3+^, and Co^2+^ replace the trivalent aluminium with C_2_ node symmetry. As a result of this substitution, three magnetically non-equivalent EPR spectra are observed, which repeat after 120° when the magnetic field is rotated in a plane perpendicular to the C_3_ axis. The nearest surroundings of the Al^3+^ ion are six oxygen ions forming a deformed octahedron. The *Z*-axes of the EPR spectra are slightly deviated from the C_3_ axis. In the EPR spectra of Cr^3+^ ions the angle of deviation is of the order of 2°, for Ti^3+^ −7°, for Co^2+^ −9°. On the other hand, Mn^2+^ ions replace trivalent yttrium, leading to a single spectrum with an axial symmetry corresponding to that of the D_3_. We suggest that the ratio of the radii of the host lattice ions and the admixture ions plays a major role. According to^20^, the radii of the ions have the following values in angstroms: Y^3+^ −0.9, Al^3+^ −0.53, Cr^3+^ −0.615, Ti^3+^ −0.67, Co^2+^ −0.75, Mn^2+^ −0.83. In this case, these are the ionic radii in the angstroms. The divalent manganese ions are much larger than aluminium ions and therefore substitute for yttrium ions. The divalent copper ions have an ionic radius of 0.73 and an EPR spectrum consisting of three magnetically non-equivalent positions. Based on this, it can be argued that Cu^2+^ ions replace Al^3+^ ions in the lattice and are in the deformed octahedron of oxygen ions.

The immediate surroundings of divalent copper substituted with Al3+ are formed by six oxygen ions forming a slightly deformed octahedron, as illustrated in Fig. 1a. The main member D splits into an orbital doublet Eg and an orbital triplet T2g in the crystal field of the octahedron. The lowest energy doublet is Eg. The symmetry of the node, which contains both the Al3+ ion and the Cu2+ ion that replaces it, is monoclinic with a single symmetry element, the C2 axis. The Z axes of the EPR spectra of Cr3+, Ti3+ and Co2+ point almost along the trigonal axis of the crystal, indicating a predominantly trigonal distortion of the octahedral environment of the admixture ions with a minor monoclinic contribution. In the regular octahedron, as in the distortion along the trigonal axis, the Eg doublet is not split. The monoclinic distortion together with the vibronic Jahn-Teller interaction removes the degeneracy of the orbital doublet.

In a regular octahedron, the Jahn-Teller effect leads to deformation along one of the three fourth-order C4 axes, which are rotated by 54.73° concerning the C3 axis^2^. The Z axis of the EPR spectrum of the Cu2+ ion in the studied crystals is rotated by 54(1)° concerning the C3 axis, which is strong evidence for the manifestation of the Jahn-Teller effect in the studied crystals.

Due to the monoclinic component in the crystal field potential, the three deformations along the fourth-order pseudo axis become non-equivalent. The configuration in which the deformation occurs in the direction towards the oxygen ion O1, where the Z axis of the EPR spectrum is directed, has the minimum energy.

The Eg state in a crystal field with tetragonal distortion splits into two levels with wave functions |3z2−r2> and |x2−y2>, whose energy location determines the ratio of g-factors^2^. For an elongated octahedron, g|| = 2−8λ/Δ, g⊥ = 2−2λ/Δ, where λ is the spin-orbit coupling parameter, Δ is the value of the distribution of Eg and T2g states. The experimentally determined ratio of g-factors (g|| > g⊥) in both crystals (see Table 2) shows that the main orbital is |x2−y2>.

We pay attention to Fig. 3a, b, which shows the EPR spectra of the Cu2+ ion in the YAl3(BO3)4:Cu and EuAl3(BO3)4:Cu crystals, respectively. The difference is that the hyperfine structure lines in the YAl3(BO3)4:Cu crystal have additional splitting, which is probably due to the interaction of Cu2+ electrons with the ligand nuclei.

Considering all nuclei with magnetic moment 10B μ = 1.8*μn (Sn = 3) and 11B μ = 2. 688*μn (Sn = 3/2), 27Al μ = 3.641*μn (Sn = 5/2) 151Eu μ = 3.472*μn (Sn = 5/2) and 153Eu μ = 1.533*μn (Sn = 5/2), we conclude that the ligand structure is due to 89Y μ = −0.137*μn (Sn = 1/2) nuclei. We do not observe the ligand structure in the EuAl3(BO3)4:Cu crystal because the europium nuclei are shielded from the electron cloud of divalent copper much more effectively than the yttrium nuclei in the isomorphous YAl3(BO3)4:Cu crystal. In the EuAl3(BO3)4:Cu crystal, the ligand structure is not apparent. The electron configuration of Y3+ is a closed shell of krypton [Kr] and the electron configuration of Eu3+ [Kr]4d105s26p64f6. Near the Cu2+ ion substituting for Al3+, there are two Y3+ ions at a distance of 3.65 Å, two at a distance of 5.56 Å, one at a distance of 4.15 Å, and another at a distance of 5.16 Å. The following Y3+ ions are at a distance greater than 8 Å. Determining which yttrium nuclei give rise to the hyperfine structure and the magnitude of the contribution of each nucleus can only be done by calculating the ground state electron density distribution of divalent copper.

The temperature dependence of the line width differs from similar dependences of other admixture ions located in the same lattice node as copper ions, for example, Cr3+ and Co2+ ions. The Cr3+ ion, which is found in the YAl3(BO3)4:Cu crystal, is weakly coupled to the lattice vibration and therefore the EPR line does not broaden until room temperature. In the EuAl3(BO3)4:Cu crystal, the chromium lines can only be observed up to 70 K since the spin-lattice relaxation takes place in the presence of excited states of the Eu3+ ion (322 K). The combination processes for Co2+ ions are much more efficient than the relaxation via excited states of the Eu3+ ion, so the temperature dependence of the line widths in the YAl3(BO3)4:Cu and EuAl3(BO3)4:Cu crystals do not differ. As can be seen from Fig. 4, the linewidth of Cu2+ ion increases faster with increasing temperature in the EuAl3(BO3)4 crystal, but the dependences in both crystals are described by exponential curves with almost identical exponents. A qualitative explanation can be proposed for the Jahn-Teller distortions of the immediate vicinity of the Cu2+ ion. If the divalent copper ion is located in the center of a regular octahedron, the octahedron is deformed along the 4th-order axis to remove the degeneracy of the Eg state. Since there are three such axes, we have three equivalent deformation possibilities with the same energies. This situation was first observed experimentally in^21^.

In YAl_3_(BO_3_)_4_:Cu and EuAl_3_(BO_3_)_4_:Cu crystals there is a symmetry of the Cu^2+^–C_2_ node, i.e. besides the dominant cubic crystal field there is a s hyperfine mall addition of a monoclinic component. As a consequence, the three distortions become non-equivalent and the EPR spectrum is observed only in the one with the minimum energy. The other two have higher energies and may contribute to the spin–lattice relaxation of the Cu^2+^ ion. As can be seen from the experimental dependence presented in Sect “Results”, the closest excited state in the EuAl_3_(BO_3_)_4_ crystal has a value of − 187 cm^−1^ and in the YAl_3_(BO_3_)_4_ crystal a value of − 196 cm^−1^. The situation when the Jahn–Teller effect appears in a poorly symmetric crystal is not unique, as an example the work of^22^, which describes the spectrum of divalent copper ion in a monoclinic ZnSeO_4_*6H_2_O crystal.

Along with the broadening of the EPR line with increasing temperature, a shift of the line to higher fields is observed, i.e., a decrease of *g*-factors occurs. Such a phenomenon was first observed in the spectrum of Dy^3+^ ions in KY(WO_4_)_2_ crystal^23^. Figure 5 shows the change of *g*_*z*_ from temperature for the Cu^2+^ ion in the EuAl_3_(BO_3_)_4_:Cu crystal. The change of the ground-state magnetic moment is the result of the combined action of Zeeman and orbital-lattice interactions. The temperature dependence of the EPR line shift is proportional to the value of T^4^ ${\int }_{0}^{\frac{\theta }{T}}\frac{{x}^{3}dx}{{e}^{x}-1}$ in Debye’s model^24^. Since the Debye temperature for the investigated crystal is (358 K) ^15,25^, the multiplier at T^4^ remains constant at low temperatures. When calculating the temperature shift of the EPR line, the excited states of the ion must be taken into account. The smaller the energy distance to the excited levels, the larger the temperature contribution. Figure 6 shows the fitting curve proportional to T^4^. As can be seen, it describes the observed temperature dependence of the g-factor reasonably well.

### YGa_3_(BO_3_)_4_:0.2% Cu and EuGa_3_(BO_3_)_4_:0.2% Cu

A striking feature is the appearance of a new spectrum 2 in the EuGa_3_(BO_3_)_4_:Cu crystal. The intensity of the Spectr 2 peak (Fig. 8) is maximum at 45 K. As a result, Spectr 2 is observed at excited levels that are 31 cm^−1^ away from the main level.

An explanation can be offered by considering the distortions of the complex containing divalent copper. If, in a regular octahedron, the three Jahn-Teller deformations are equivalent, then in a crystal with initial deformations due to minor contributions from the monoclinic field, at least one of the deformations will form a state that is lower in energy than the other two. It is in this state that EPR spectra with similar parameters are observed in all four crystals. The energies of the excited states in the EuAl_3_(BO_3_)_4_:Cu crystal are − 187 cm^−1^ and in the YAl_3_(BO_3_)_4_:Cu crystal are − 196 cm^−1^. In the YGa_3_(BO_3_)_4_:Cu crystal, it was not possible to determine the energy of the excited levels by broadening due to the initially large line width. In the EuGa_3_(BO_3_)_4_:Cu crystal, the excited state has an energy of 31 cm^−1^, as a result of which an unusual thermal behavior of Spectr 2 is observed. When the temperature is further increased above 70 K, a broad isotropic line appears as a result of averaging of the excited states.

## Conclusion

The investigation of europium and yttrium aluminum borates (EuAl_3_(BO_3_)_4_ and YAl_3_(BO_3_)_4_, respectively), as well as gallium aluminum borates (YGa_3_(BO_3_)_4_ and EuGa_3_(BO_3_)_4_), doped with copper impurities was conducted. The crystalline structure of the studied materials was determined using X-ray diffraction methods. It was found that copper ions are in a divalent state and substitute for aluminum or gallium ions in the investigated crystals. As a result of the manifestation of the Jahn-Teller effect, the nearest environment of the copper ion is distorted in such a way that the magnetic axis *Z* of the Cu^2+^ ion in the studied crystals is rotated relative to the C_3_ axis by 54(1)°.

The parameters of the EPR spectrum were determined—*g*-factors and hyperfine splitting constants. Each hyperfine line in the YAl_3_(BO_3_)_4_:Cu crystal has additional splitting due to yttrium nuclei (*I* = 1/2). In the EuAl_3_(BO_3_)_4_:Cu crystals, the ligand hyperfine structure is not observed due to the more effective shielding of europium nuclei by its electron shell. With increasing temperature above 20 K, spectral line broadening occurs due to an increase in the spin–lattice relaxation rate, which is described by an exponential dependence.

In the EuGa_3_(BO_3_)_4_:Cu crystal, a new Spectr 2 belonging to divalent copper was detected, but it is observed in an excited state, shifted by 31 cm^−1^ from the ground state. With increasing temperature, an isotropic broad line characteristic of the Jahn-Teller effect appears, which is caused by the averaging of excited states.

## Methods

### Synthesis

Crystals of YAl_3_(BO_3_)_4_ and EuAl_3_(BO_3_)_4_ doped with 0.1% Cu^2+^ were obtained by spontaneous crystallization from a molten solution. The composition was thoroughly mixed to achieve a homogeneous mass, then placed in a platinum crucible and heated to 750 °C for 5 h. Potassium molybdate, K_2_Mo_3_O_10_, was used as the solvent. Growth took place in platinum crucibles by cooling the solution from 1150 to 900 °C at a rate of 2 °C/h. The investigation was conducted on well-faceted crystals with dimensions of 2–3 mm.

Crystals of YGa_3_(BO_3_)_4_ and EuGa_3_(BO_3_)_4_ doped with 0.2% of copper were obtained by the similar crystallization method. A solvent mixture of Bi_2_O_3_ + B_2_O_3_ was chosen and cooled from 900 to 700 °C at a rate of 2 °C/h. Transparent faceted crystals with sizes of 0.5–1 mm were obtained.

### XRD investigations

The crystalline structure of the materials being the object of investigation was determined by X-ray powder diffraction method (XRD). The XRD measurements were done with help of a X’Pert Pro Alpha1 MPD laboratory diffractometer equipped with a Cu X-ray tube, a primary beam Johansson type monochromator (Ge (111)) and a semiconductor detector (X’Celerator). The Rietveld method realized by Fullprof.2 k program^26^ was applied for analyzing collected diffraction patterns. A set of refined parameters based on the crystallographic model of investigated crystals and geometry of XRD measurements have been enforced.

### Electron paramagnetic resonance

The EPR spectra were measured using X and Q -band radiation, within the temperature range of 5–500 K. Continuous wave EPR spectra were measured by using a Bruker X-/Q -band E580 FT/CW ELEXSYS spectrometer. For the measurements, the ER 4122 SHQE Super X High-Q cavity with TE011mode was used. The samples were placed into quartz rods of 4 and 2 mm in diameter. The experimental parameters were: micro-wave frequency 9.8 GHz, 37 GHz microwave power 1.500 mW, modulation frequency 100 kHz, modulation amplitude 0.2 mT, and the conversion time 60 ms.

## Data Availability

No datasets were generated or analysed during the current study. The data that support the findings of this study are available from the corresponding author upon reasonable request.
